# Vibration-Based Machine Learning Model Training for Railway Bridge Health Monitoring †

**DOI:** 10.3390/s26134323

**Published:** 2026-07-07

**Authors:** Rocco Alaggio, Muhammad Asad, Riccardo Cirella, Stefania Costantini, Giovanni De Gasperis

**Affiliations:** 1 Dipartimento di Ingegneria Civile, Edile—Architettura e Ambientale, Università degli Studi dell’Aquila, 67100 L’Aquila, Italy; riccardo.cirella@graduate.univaq.it; 2 Dipartimento di Ingegneria e Scienze dell’Informazione e Matematica, Università degli Studi dell’Aquila, 67100 L’Aquila, Italy; muhammad.asad@graduate.univaq.it (M.A.); stefania.costantini@univaq.it (S.C.); 3 School of Computer and Information Technology, Beaconhouse National University, Lahore 53700, Pakistan

**Keywords:** accelerometer, anomaly detection, machine learning (ML), neural network, steel bridges, structural health monitoring

## Abstract

Bridge health monitoring and machine learning are increasingly intertwined for civil engineers and artificial intelligence experts. Bridges’ poor health can result in severe outcomes if not addressed in time. Therefore, continuous monitoring is required to detect any anomaly or damage. Sensors, such as accelerometers, inclinometers, thermistors, etc., can help actively monitor these bridges. The signals from these sensors help record physiological activities. Such activities are helpful for anomaly detection, damage localization, and bridge health predictions with the help of machine learning algorithms. The proposed method extracts features from the dynamic response of a bridge to ambient excitation. It focuses on processing the signal received from different accelerometers installed on a steel railway bridge to determine the location of the damage and the level of the damage predictions. Initially, features are extracted from time-series data; then, they are fed to a deep neural network after some pre-processing. Normal and augmented data are used with different parameter tuning for results. Original data is also subdivided, and the effect of data slicing on the predictions is investigated. The results show that one-fourth of the slicing of the original data gives the best results for training and testing accuracy with a deep neural network. The results show that the reduced matrix representation, particularly the 40 × 40 feature slicing, improved the classification performance for the predefined bridge scenario classes under the considered experimental settings. For bridge scenario classification, the best reported accuracy was 93.54%, while for damage intensity classification the best reported accuracy was 98.21%. In the DNN-based optimizer comparison, the Adam optimizer achieved higher and more stable performance than Stochastic Gradient Descent (SGD), with test accuracies of 92.3% and 93.7% compared with 75.2% and 86.4%, respectively. It is also observed that the Adam optimizer outperformed Stochastic Gradient Descent (SGD) in terms of both damage localization and damage intensity estimation.

## 1. Introduction

Structural health monitoring (SHM) of bridges has become an increasingly important focus. What prompted research to explore this topic more thoroughly is the recognition that structures, during their life, are subject to aging or degradation phenomena. These phenomena may result in a loss of structural performance, which ultimately leads to the inability of the structure to fulfill its intended functional requirements. The strategic importance of bridges as critical elements for mobility and logistic infrastructure makes their monitoring even more essential than ordinary structures. Any potential compromise of their integrity could lead to significant disruptions in transportation systems and severe economic and social impacts.

The assumption at the base of damage detection is that when any form of damage or degradation occurs, the structural parameters change, and consequently, the response of the structure is altered as well: the objective of monitoring a structure is, therefore, to detect this behavioral change and to investigate its causes.

SHM involves continuously evaluating the bridge’s condition, assessing progression of damage, and timely maintenance interventions.

In this context, a structural monitoring system can be helpful in two different phases of a structure’s life: during maintenance, to focus interventions on parts of the structure where anomalies emerge, and during emergencies, as a management tool for selecting intervention priorities.

Damage detection techniques can be categorized into four stages, as outlined by Rytter [1] and developed over time: identification, localization, quantification of damage intensity, and prognosis. In line with these levels, many techniques have been developed over time. Prior to machine learning-based approaches, several classical techniques for vibration-based SHM were developed. These vibration-based techniques are related to structural damage detection, localization, and quantification. These methods mainly rely on changes in model parameters, since damage usually modifies the stiffness distribution of a structure. Early studies used variations in natural frequencies for damage identification [2,3], while later works showed that mode shapes and their spatial derivatives provide stronger indicators for localizing damage. In particular, changes in mode shape curvature have been used, as it is more sensitive to local stiffness reduction than the mode shape [4].

Other stable approaches include modal flexibility methods [5], modal strain energy-based damage indices [6], frequency response function-based indicators, and general vibration-based SHM frameworks [7,8,9]. These conventional techniques provide a strong reference foundation for structural damage monitoring. These methods also remain important benchmarks for evaluating modern data-driven and machine learning-based damage detection techniques [10].

Recently, the development of artificial intelligence techniques has driven the research community’s interest in applying machine learning algorithms to solve damage identification and classification problems. Most use non-destructive methods that involve sensor data acquisition (accelerometers, crack gauges, etc.) placed at significant locations of the structure [11].

Neural network-based techniques for bridge monitoring [12,13,14,15,16,17,18], including our previous work about bridge health monitoring using the RBF-Neural Network [12,19] method for bridge SHM, prove to be more cost-effective, faster, and more accurate. Most of these works focus on time-domain- or frequency-domain-based implementations.

Nowadays, engineers analyze and monitor structures in real time to identify anomalies and achieve early detection of damages. They also ensure they take proactive maintenance measures and extend the structural lifespan of such bridges.

Anomaly detection [17], damage localization [13,20,21], damage classification [15], monitoring response, and alert generation based on structural factors are becoming easy to investigate in steel bridges with machine learning algorithms. However, a lot of work is still needed to address specific challenges. These challenges include technological difficulties, environmental factors, maintenance cost, socioeconomic balance, and risk to safety [22].

This study covers a damage identification analysis of railway steel bridges using a deep neural network with different parametric tuning and the effect of data slicing on performance matrices. The objective is to use features extracted from vibrational signals to train the networks to identify and classify structural damage.

It is important to note that, when dealing with complex structures such as those in civil engineering, the damage classification phase (i.e., the Rytter scale levels above the first) necessarily requires the development of a mathematical model of the structure itself. This model is essential for estimating structural behavior under potential damage scenarios.

The proposed approach uses the structure’s physics-based reduced-order model (ROM) to generate a dataset of expected sensor measurements corresponding to various asset states with damage at different locations and intensities. This dataset is then used to train a classifier capable of processing near-real-time sensor data and estimating the asset’s underlying state.

The methodology involves an initial Data Dimensionality Reduction (DDR) step, where accelerometric time-series data are processed to form block Toeplitz correlation matrices. These matrices are obtained from the block Hankel matrix of output data, the so-called subspace matrix, in the Data-Driven Stochastic Subspace Identification (DD-SSI) technique [23], widely employed in civil engineering for Operational Modal Analysis. Block Toeplitz matrices are then used with neural networks for damage classification. The classification identifies both the location and the intensity of the damage.

The paper is organized as follows. Section 2 explains some background studies and previous research carried out in this domain; Section 3 outlines the objectives of the present work and how they have been pursued; and Section 4 covers dataset generation, pre-processing and feature extraction, and data augmentation. Section 5 presents the research methodology, and Section 6 presents the observed results. The paper concludes with a discussion about future directions.

## 2. Background

In recent years, non-destructive structural damage detection techniques based on structural dynamic characteristics, genetic algorithms, and ANNs have been developed. Inspired by these methods, AI experts have been interested in applying deep neural networks in bridge health monitoring.

Thanh et al. [21] analyzed the non-destructive SHM of bridges using the fusion of Particle Swarm Optimization and Support Vector Machines (POS-SVM). POS-SVM helped eliminate the redundant input parameters for adequate classification of damage localization. Similarly, in [24], a supervised method based on SVM was introduced, using auto-regressive feature extraction methods for steel bridges. Elisa et al. [18] used an ANN for anomaly detection and damage localization in time-series data for structural health monitoring. They focused on recording the axle load signal every time a train passed over the bridge. Khodabendelou et al. [25] designed a feedforward CNN to evaluate bridge health using vibration response, and Abdeljaber et al. [26] carried out optimization for non-parametric structural damage detection for performance evaluation using a CNN with a single learning block for training.

In [27], Huile et al. also examined railway bridges for structural health monitoring and damage detection. They analyzed the railway bridge girder for Chinese high-speed rail, using a Bayesian deep learning method to account for dynamic speed loading. Abdelkader et al. [28] investigated damage detection and localization with real-time vibration data using a simple CNN. This model can automatically extract optimal damage-sensitive features from raw acceleration signals. Another study by Giasi et al. [29] adopted a CNN for damage classification on railway steel bridges using various damage parameters. They also managed to achieve accuracy using both accurate data and Finite Element model data.

Several machine learning-based structural health monitoring (SHM) studies have explored diverse modeling techniques, feature extraction methods, normalization strategies, and evaluation metrics. For instance, ref. [24] applied supervised learning using K-nearest neighbors (KNN), Support Vector Machines (SVMs), Gaussian Naive Bayes (NB), and Random Forests (RFs). They employed auto-regressive features, normalized the data using the Mahalanobis Squared Distance, and evaluated performance using ROC curves and confusion matrices. Ref. [30] used Artificial Neural Networks (ANNs) with Singular Value Decomposition (SVD) for feature extraction and RMSs for normalization, assessing performance in terms of damage intensity accuracy. On the other hand, refs. [31,32] adopted unsupervised approaches. While ref. [31] focused on dynamic and static response changes without specifying feature extraction or performance metrics, ref. [32] utilized a threshold-based SVD classification approach with SVD, Least Squares Vectors (LSVs), and Independent Component Analysis (ICA) for feature extraction. They normalized data using the Euclidean Norm and evaluated performance through novelty index comparison, outlier analysis, and novelty detection accuracy. Ref. [14] implemented supervised learning combining model order reduction and fully convolutional neural networks (CNNs), using Principal Component Analysis (PCA) for feature extraction and evaluating performance based on accuracy and signal-to-noise ratio. Ref. [33] integrated linear regression with robust PCA, using PCA for features and normalizing via the Mahalanobis Distance and the Chi-Square Distribution, with ROC used for evaluation. Ref. [21] used a supervised approach involving a POS-SVM model with feature selection via the genetic algorithm (GA), and root mean square error (RMSE) as the evaluation metric. Ref. [18] deployed ANN models, normalized data using the mean and standard deviation, and evaluated results using ROC, true positives (TPs), and false positives (FPs). Lastly, ref. [29] utilized a CNN-based model with t-distributed Stochastic Neighbor Embedding (t-SNE) and Gradient-weighted Class Activation Mapping (Grad-CAM) for feature extraction, Gaussian noise for data normalization, and accuracy as the performance measure.

Collectively, these studies highlight the range of methodologies used in SHM, demonstrating the adaptability of both traditional and deep learning approaches, various dimensionality reduction and visualization techniques (e.g., PCA, t-SNE, SVD), and performance evaluation strategies tailored to damage detection and condition assessment in structural systems.

## 3. Research Questions

This study aims to evaluate the performance of a machine learning approach for the classification of structural damage in railway infrastructures. The issue of damage classification has been and continues to be the subject of extensive research in civil engineering due to the complexity of predicting structural behavior in the presence of damage.

As mentioned in the previous section, it is impossible to produce a realistic damage condition reversibly when working with existing structures. Therefore, implementing a physical–mathematical model to create simulated data is crucial when characterizing structure damage. This study adopts a ROM model of a railway bridge to produce the data necessary for training and testing a CNN for damage classification.

The CNN is designed to answer the following research questions:

- Is it possible to use the dynamic subspace matrix to extract features useful for damage classification through ML techniques?
- Can the feature matrix slicing size affect the classification problem?
- What would be the effect of different optimizers on the accuracy of the bridge section and damage intensities?
- What would be the effect of data augmentation on the final results?

These research questions are explored in subsequent sections.

## 4. Dataset Description

**Figure 1:**
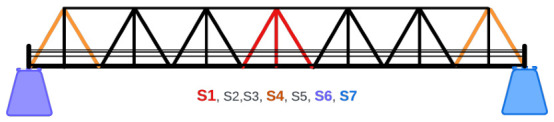
Side view of the bridge model. The colored regions indicate the predefined damage scenario groups, labeled S1–S7, corresponding to selected structural element groups in the FE model.

**Figure 2:**
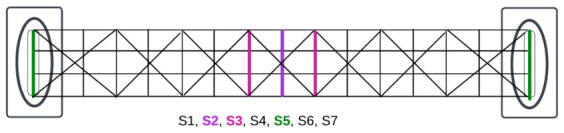
Aerial view of the bridge model. Different colors represent the predefined damage scenario groups S1–S7, used as damage-location classes in the dataset. Each colored region corresponds to a selected group of structural elements considered for simulated damage generation.

The model is based on the geometry and mechanical characteristics of an existing steel railway bridge near Città di Castello, Italy. The structure is made up of three steel reticular beams, which allow the railway network to cross the underlying Tiber River: plane and elevation views of the central span can be seen in Figure 1 and Figure 2. The highlighted colors represent the group of accelerometer sensors installed at each of seven bridge scenarios, i.e., S1, S2, S3, …, S7.

Recently, a vibration monitoring system was installed on the central span of the structure. The system consists of six accelerometer sensors, mostly uni-axial in the vertical direction. In the centre of the span, there are bi-axial sensors, additional components oriented in the transversal direction of the bridge. Therefore, there are a total of eight measurement channels. The accelerometers are very low-noise, high-dynamic Force-Balance-type, and are connected via cables in two synchronized chains: signals are collected with a sampling frequency of 200 Hz. A GPS receiver on each data recorder allows the data coming from the two chains to be synchronized in time. The choice of very low-noise sensors allows the system to record even slight vibrations, such as those generated by the surrounding environment of the bridge.

The signals are recorded under ambient noise conditions (train passages are not collected for this study).

Starting from the acquired ambient vibration series and considering the geometry of the bridge, the model was developed to replicate the actual structure’s dynamic behavior optimally. For this purpose, the model’s parameters were optimized through a parametric calibration process, whose detailed description is beyond the scope of this study.

The model was then used to simulate the structural response in different damage scenarios.

The damage is generated by selecting specific elements of the FE model, whose positions are pre-determined and labeled by alphanumeric symbols (from S1 to S7). It consists of the reduction in the mechanical property of the elastic modulus in proportion to the chosen damage intensity level. Intensity levels are represented by a percentage value ranging from 1% to 90%: a more detailed description of the scenarios is provided in Table 1.

Therefore, the dataset used for training the neural network is built starting from the time series generated by the FE model for each damage scenario and intensity. The time series are recorded at the nodes of the FE model in the same position as the sensors placed on the real structure. Each simulation produces eight time series lasting 30 min, sampled with a frequency of 200 Hz, for a total of 360,000 samples per series.

Additionally, for each damage scenario and intensity, the simulation is repeated 180 times, varying the excitation applied to the model.

In particular, the excitation was applied to the FE model as a dynamic force input at the predefined excitation location(s), while the structural responses were recorded at the nodes corresponding to the sensor locations on the real structure. Across the 180 simulations, the excitation was varied in terms of its amplitude and time-history characteristics in order to generate different vibration response realizations for the same damage scenario and damage intensity. The 26 damage intensity levels were treated as damage-definition parameters rather than excitation parameters.

The time series produced are then processed in the DDR phase: the correlation matrices are obtained, whose dimensions are 320 × 320, a number proportional to that of channels and to the inner parameters of the applied technique. These matrices are then used in feature extraction to train and test ML classifiers, as described in the following section.

## 5. Methodology

This section describes the detailed methodology and implementation of a deep neural network framework. This framework is used to detect multiclass-based behavioral anomalies in different sections of a steel bridge and to monitor its health, assessing the severity of the damage at various intensity levels.

This section also introduces the features extracted from the simulated acceleration data used in the CNN training. Furthermore, it also discusses the machine learning algorithm, its evaluation using standard data and augmented data, fine-tuning of deep neural network parameters, model parameters, and finally, the evaluation criteria for damage localization and damage level classification. Therefore, this section is divided into four subsections, namely, (i) Basic Framework, (ii) Feature Engineering, (iii) deep neural network algorithm, and finally, (iv) evaluation criteria.

### 5.1. Basic Framework

**Figure 3:**
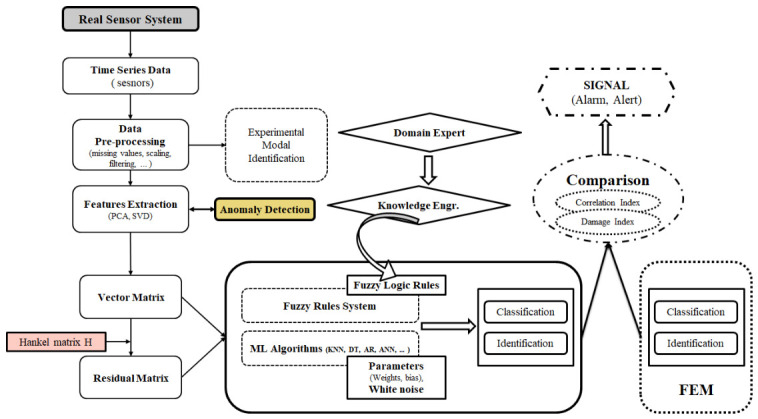
Final deployment framework of railway bridge health monitoring system. The colour legend indicates that grey represents real sensor data, orange represents physics-based FEM data and pink represents sensory data converted using a Hankel matrix and statistical methods to extract features.

**Figure 4:**
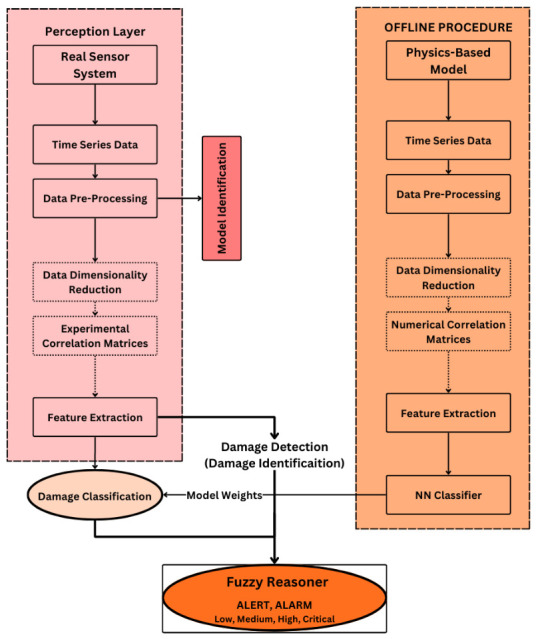
Training process schema for damage classification.

The damage characterization procedure is part of a broader bridge health monitoring workflow, as illustrated in Figure 3. In this workflow, vibration responses from the bridge are processed to extract damage-sensitive features, which are then compared with features generated from the physics-based calibrated numerical model. Once an anomalous condition is detected, the diagnostic stage uses the trained classifier to estimate the corresponding predefined bridge scenario and damage intensity class. Figure 4 summarizes the training process used in this study. In the present work, the experimental evaluation is limited to classification of predefined bridge scenarios and damage intensity classes; therefore, any higher-level reasoning or decision-support module is considered part of the future deployment framework and is not included in the reported performance evaluation.

The damage characterization (bridge scenario and damage intensity) as discussed in the previous sections is part of a broader bridge health monitoring workflow, as illustrated in Figure 3. Figure 4 summarizes the training process schema used in this study. In the present work, the experimental evaluation is limited to classification of seven predefined bridge scenarios and 26 damage intensity classes; therefore, any higher-level reasoning or decision-support module is considered part of the future deployment framework and is not included in the reported performance evaluation.

Physics-based modeling is required in the diagnostic phase when investigating the location and intensity of the anomaly; after that, its presence has been verified. In this phase, the current state of the structure is classified by comparing its features with those from a trained neural classifier. Based on the information provided during the training phase, the network predicts classes relative to the damage scenario and intensity; see Figure 4.

The same framework can be used in both the time and frequency domains, even if the study focuses only on time-domain analysis. Thus, the system implementation includes a Fuzzy Logic Reasoning Layer with anomaly detection, a DNN for classification, and damage localization based on features extracted from time series. Bridge scenarios and damage intensities are prediction classes produced by the neural perception layer. Machine learning-based algorithms with standard data and augmented data are used for predictions. For bridge scenarios, seven classes are referred to that a bridge can be categorized as for analysis or monitoring purposes. Similarly, twenty-six classes are called damage intensities in the range of 0–100%. Each damage intensity is calculated by applying specific damage to the model, as described in the previous section. The damage intensities are a set of discrete intervals. Domain experts then converted them into compact classes with labels of low, medium, high, and extreme damage intensities.

Before feeding the matrix data to a DNN, it is sliced to 100%, 50%, 33%, and 25%. This slicing is carried out to check the effect of such slicing on original data regarding prediction accuracy. The other purpose of this way of slicing is to maximize the physical data of the bridge as much as possible. To enhance data availability, the original 320 × 320 dataset was first flattened into a one-dimensional sequence and then restructured into matrices of sizes 180 × 180, 80 × 80, and 40 × 40 by sub-sampling so that the transformation preserves the temporal and spatial dependencies within the data by leveraging its inherent spatial continuity, ensuring that meaningful structural relationships remain intact. Consequently, the model can effectively capture local and global patterns, essential for an accurate analysis. By increasing the size of the dataset through matrices obtained with structured sub-sampling, this approach enhances the model’s generalization ability, improving its robustness and reliability in real-world applications. These sliced data are then converted into features as explained in Section 5.2.

### 5.2. Feature Engineering

Accelerometer sensors are installed at different locations on the bridge. There are mono-axial, bi-axial and tri-axial accelerometer sensors. Mono-axial sensor data consist of X-axes, bi-axial accelerometer data consist of X-, and Y-axes and tri-axial accelerometer data consist of X-, Y-, and Z-axes (vertical, lateral, and sagittal axes) [34]. These sensors are used to monitor the train’s activity over the bridge. The train activity on the bridge helps monitor the structural health of the bridge.

Data received from the accelerometer sensor are in raw form. Data received in the time domain are then converted to features. In addition, to clean the data, a pre-processing step is performed. This pre-processing step is applied to obtain valuable ambient features, which are later fed to machine learning models for anomaly detection, damage localization, and predictions. The cumulative sum of differences [35], cosine similarity [36], Skewness [37,38], Kurtosis [37,38], energy [39], and crest factor [40], etc., are the calculated ambient features. Principal Component Analysis (PCA) is then applied for feature selection. Five features are selected with PCA; other features, which have low correlation values, are discarded. Therefore, only selected features after PCA are explained here.

#### 5.2.1. Normal Features

Initially, ambient features are calculated using the data from the original accelerometer sensor. The following dataset features are reported in bold and explained.

**Cumulative sum of differences** is calculated using the formula given in (1). It is the sum of the difference between the original reference value and the relevant subsequent values received from the sensor. A logarithmic transformation is applied to the final values as these values are too small.

(1) S=log(∑|b−a|)

As discussed earlier, a reference data matrix is assigned to calculate the features. The 1st matrix of each damage intensity is used as a reference matrix. Here, ‘a’ is used as an element of reference matrix ‘A’. ‘b’ represents the elements of all other respective matrices ‘B’, which is used to get a cumulative sum of differences by subtracting them from the reference matrix ‘a’ and calculating their sums. After analyzing the data, the log function is applied to normalize the generated data.

**Crest factor (CF)** (3) is calculated in terms of the peak value-to-root mean square (RMS) ratio. The crest factor indicates the extreme peaks of a waveform,

(2) $$CF=\frac{peak\_value}{RMS}.$$

Equation (2) thus becomes (3) for the crest factor (CF) after inputting the matrix data.

(3) $$CF=\frac{\max(|\mathrm{matrix}|)}{\sqrt{\frac{1}{N}\sum_{i=1}^{N}{\left(\mathrm{matrix}\left[i\right]\right)}^{2}}}$$

**Cosine similarity (CS)** is a similarity measure between two non-zero vectors defined in an inner product space. Cosine similarity is the cosine of the angle between the vectors; that is, it is the dot product of the vectors divided by the product of their lengths.

(4) $$CS\left(a,b\right)=\frac{\sum_{i=1}^{n}a_{i}\cdot b_{i}}{\sqrt{\sum_{i=1}^{n}a_{i}^{2}}\cdot \sqrt{\sum_{i=1}^{n}b_{i}^{2}}}$$

**Energy** is considered as the strength of a signal over some time interval. It quantifies the signal’s magnitude. The discrete signal is calculated using Equation (5) instead of a continuing signal method. In the last step, a logarithmic transformation is applied to normalize the values.

(5) $$E=log(\sum_{n=n_{1}}^{n_{2}}|x\left[n\right]{|}^{2})$$

The logarithmic function is also applied to the crest factor and cumulative sum of differences values as these values are too small to handle easily.

#### 5.2.2. Data Augmentation

After converting the signal data into ambient features, data augmentation is applied. Data augmentation is performed using Gaussian normal distribution methods within the range of ‘0’ and the standard deviation of the data. This can be expressed by (i) noise generation, (ii) data augmentation, and (iii) range checking and correction, which are reported in bold with their definition:

**Noise Generation:** a random noise (6) is generated from a Gaussian normal distribution with a mean of ‘0’ and the standard deviation *(noise_std)* of data gathered from a specific sensor. This can be represented as

(6) noise∼N(0,noise_std)

**Data Augmentation:** the noise generated is added to the selected feature columns of the input data as augmented data (7). If the original data is denoted as *‘data’* and the selected feature columns as *‘feature_columns’*, the augmented data *‘augmented_data’* is obtained as

(7) augmented_data=data+noise

**Range Checking and Correction:** Equation (8) checks if any values in the augmented data are outside a valid range. Suppose that a value in the augmented data is less than 0 or greater than the standard deviation of the corresponding feature column in the original data. In that case, it is replaced with the original value from the input data. Otherwise, the original augmented data value is used. Mathematically, this step can be represented as

(8) $$\mathrm{aug}\_\mathrm{data}\left[i\right]=\left\{\begin{array}{cc} \mathrm{data}[i] & \mathrm{if}\;\mathrm{aug}\_\mathrm{data}[i]<0\;\mathrm{or}\\ \; & \mathrm{aug}\_\mathrm{data}[i]>\mathrm{std}\_\mathrm{dev}\_\mathrm{fts}[i]\\ \mathrm{aug}\_\mathrm{data}[i] & \mathrm{otherwise} \end{array}\right.$$

Here, *std_dev_fts* represents the standard deviation of the corresponding feature column in the original data. These mathematical representations help illustrate the operations performed in data augmentation with Gaussian noise and ensure the augmented data remain within a valid range.

In this study, Gaussian noise-based augmentation is applied to features to increase the training data variability. It should also be noted that data augmentation is not intended to represent the physics-based model of sensor noise, ambient uncertainty, or vibrational variability. Instead, this Gaussian noise-based data augmentation is applied as a controlled data augmentation strategy to improve the robustness of the classifier under small variation in the extracted features. Therefore, after adding noise, range checking is applied to avoid augmented values outside the admissible feature range and it also preserves consistency with the original feature distribution. The effectiveness of this Gaussian augmentation strategy was evaluated by comparing the classification performance obtained with original data as well as with augmented data.

### 5.3. Classical Machine Learning Models

Before the rise of neural network models, traditional machine learning models played an essential role in foundational methods. These models, KNN [41], SVM [42], DT [43], RF [44], Ensemble Learning [45], and MLP [46,47], are widely used for supervised learning and unsupervised learning tasks for classification, regression, predictions, and clustering.

### 5.4. DNN Algorithm

It is a special class of neural network (NN) models [48] that is frequently used and has shown good performance in computer vision and image processing tasks, such as image processing, object detection, image segmentation and localization [49]. A CNN generally consists of several layers of digital neurons comprising a deep NN architecture: fully connected layer (FC), pooling layer, activation function, batch normalization, dropout, and softmax (classification layer). Several different parameters can be optimized in each layer, and each layer performs a specific task with input data from the previous layer. The Conv layer has a set of filters applied to the input data to extract features. Equation (9) shows the essential convolutional operation in the CNN model.

(9) $$y_{j}=\sum_{i=1}^{n}w_{ij}x_{i}+b_{j}\;\mathrm{for}\;j=1,2,\ldots ,m$$

where $x=[x_{1},x_{2}\ldots ,x_{n}]$ is input vector of size *n*, $y=[y_{1},y_{2}\ldots ,y_{m}]$ is the input vector of size *m*, and $w_{ij}$ is the weight connecting input *i* to output *j*. In contrast, $b_{j}$ is the bias for the jth output and $y_{j}$ is the jth output. In matrix form,

(10) y=Wx+b

where **W** is the weight matrix of size m∗n and **b** is the bias vector of size *m*.

**Figure 5:**
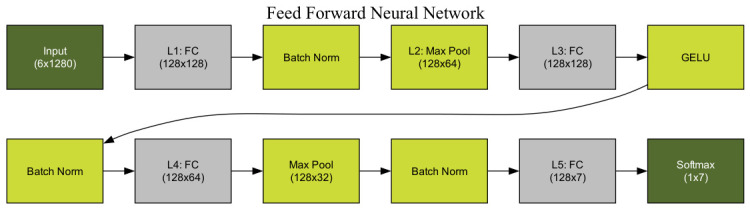
D-Net architecture for our bridge health monitoring system. A 5-layer neural network processing 6 × 1280 input tensors through alternating fully connected, batch normalization, max pooling, and GELU activation layers. The network outputs a 1 × 7 probability distribution via Softmax. FC = Fully Connected; GELU = Gaussian Error Linear Unit.

The implemented model is a deep neural network (DNN), as shown in Figure 5. This DNN has ambient features as input, multiple hidden layers, and a softmax output for seven-class and six-class predictions. Equation (11) shows our linear neural network’s essential 1D convolutional operation. Let us discuss the internal architectural breakdown of the classified DNN.

(11) $$y\left(t\right)=\sum_{i=0}^{k-1}x\left(t\cdot s+i\right)\cdot w\left(i\right)$$

where *t* is the output index (corresponding to the window position on the input), and *s* is the stride, which determines how much the window shifts at each step. x(t·s+i) is input data within the window and w(i) kernel weights.

Architecture: the model consists of fully connected (linear) layers. There are no convolution layers characteristic of convolution neural networks (CNNs). Table 2 summarizes our deep neural network’s hyper-parameters and architecture.

One-Dimensional data handling: as feed, input (R × 7) and (R × 4) for the prediction of 7 classes and 4 classes reduced from the original 26 classes, respectively. The model appears to operate on 1-dimensional data given the presence of 1-D Max-pooling.

Activation Functions: the model uses activation functions such as Leaky-ReLU and GELU, which are commonly used in DNNs. GELU is widely used in DNNs to introduce non-linearity into the model, enabling it to learn complex relationships in the data.

The Gaussian Error Linear Unit (GELU) activation function is defined as

(12) GELU(x)=x·Φ(x)

where Φ(x) is the cumulative distribution function (CDF) of the standard Gaussian distribution.

(13) $$\Phi \left(x\right)=\frac{1}{2}\left[1+\mathrm{erf}\left(\frac{x}{\sqrt{2}}\right)\right]$$

with approximation defined by

(14) $$\mathrm{GELU}\left(x\right)\approx \frac{x}{2}\left[1+\tanh\left(\sqrt{\frac{2}{\pi }}\left(x+0.044715x^{3}\right)\right)\right]$$

Gaussian Error Linear Unit (GELU) activation introduces smoothness and probabilistic behavior to neural networks with the erf(x) error function, defined as

(15) $$\mathrm{erf}\left(x\right)=\frac{2}{\sqrt{\pi }}\int_{0}^{x}e^{-t^{2}}\;dt$$

The GELU activation function determines the output by weighing the input *x* with the probability that it is greater than a random variable drawn from a standard Gaussian distribution. It introduces smooth non-linearity and is particularly effective in tasks involving transformers and attention mechanisms. The cumulative distribution function Φ(x) ensures that the output transitions smoothly between linear and non-linear regions. The approximation simplifies computation while retaining the core probabilistic behavior of the original GELU. In addition to GELU, we introduced Leaky-ReLU in our fully connected neural network. Leaky ReLU is a standard ReLU activation function variation that allows a slight, non-zero gradient when the input is negative. This helps avoid issues such as the dying ReLU problem, where neurons become inactive during training.

The Leaky Rectified Linear Unit (Leaky ReLU) is an activation function that introduces a slight slope for negative inputs, helping to prevent the dying ReLU problem. It is defined as

(16) $$\mathrm{Leaky}\;\mathrm{ReLU}\left(x\right)=\left\{\begin{array}{cc} x, & \mathrm{if}\;x>0\\ \alpha x, & \mathrm{if}\;x\leq 0 \end{array}\right.$$

Here, α is a small positive constant, typically α=0.01. For x>0, the function behaves as standard ReLU.

(17) LeakyReLU(x)=x

For x≤0, the function has a small slope controlled by

(18) α:LeakyReLU(x)=αx

The main advantage of Leaky ReLU is that it avoids the issue of neurons becoming inactive during training by allowing a slight gradient even for negative values of *x*. Our neural network model used GELU and Leaky-ReLU after the second and third fully connected layers.

Batch Normalization: while batch normalization is also used in CNNs, its presence does not necessarily indicate a CNN, especially considering the absence of convolution layers. Batch normalization is also used to improve training stability and convergence. Batch normalization is commonly used to normalize the activation of each layer, leading to faster training and better generalization. We used four batch normalization layers, each preceding a fully connected layer.

Output Layer: The model ends with a softmax activation, typical in DNNs, for multiclass classification tasks.

The softmax function converts a vector of numbers into a probability distribution. It is commonly used in machine learning, particularly in the output layer of a neural network used for classification tasks.

The softmax function for a vector $z=[\;z_{1},z_{2},\ldots ,z_{n}]$ is defined as

(19) $$\sigma {\left(z\right)}_{i}=\frac{e^{z_{i}}}{\sum_{j=1}^{n}e^{z_{j}}}$$

where $\sigma {\left(z\right)}_{i}$ is the *i*-th component of the output vector, $z_{i}$ is the *i*-th component of the input vector z, *n* is the number of elements in the input vector z, and *e* is the base of the natural logarithm. As we deal with damage intensities and bridge scenarios, we have 26 softmax outputs for damage intensities and a 7-class softmax output for bridge scenarios.

In conclusion, the provided DNN model suits required tasks such as steel bridge damage detection and classification, making up the perception layer sub-system in the overall bridge monitoring software architecture.

## 6. Results

ML models (SVM, MLP, RF, KNN, Ensemble (KNN, RF, and SVM), and DNN) were implemented using Pytorch 2.0 and Python 3.9 for programming purposes using the NVIDIA 940MX GPU. As a model optimization kit, a DL framework, the back-end for detecting anomalies and classifications, was used to prepare, train, and optimize the model. All experiments were performed on the Jupyter Notebook, version 7.5.5, platform using an NVIDIA GPU with 16 GB memory.

### 6.1. Bridge Scenario Predictions

The model’s performance was assessed across the dataset explained above. The dataset was divided into training and testing using an 80–20% ratio, respectively. We initially used basic machine learning algorithms for performance evaluations, including KNN, SVM, Random Forest, MLP, and an Ensemble of KNN, RF, and SVM with majority rule voting. Later, we also developed our own DNN model for performance evaluation. Detailed performance evaluations based on machine learning and DNN are discussed below.

#### 6.1.1. Machine Learning Algorithms to Classify Damage Scenarios

**Figure 6:**
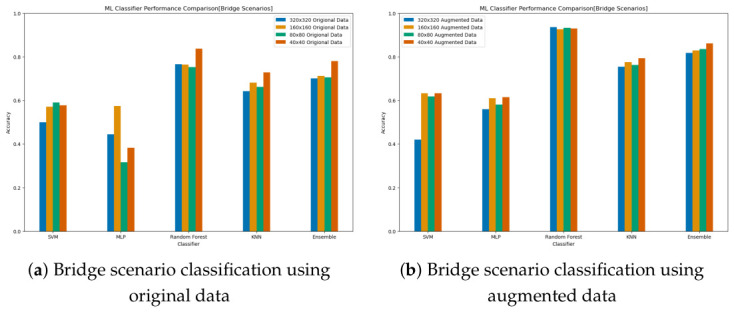
Performance comparison of traditional machine learning models for bridge scenario classification using original and augmented data across different matrix sizes.

**Figure 7:**
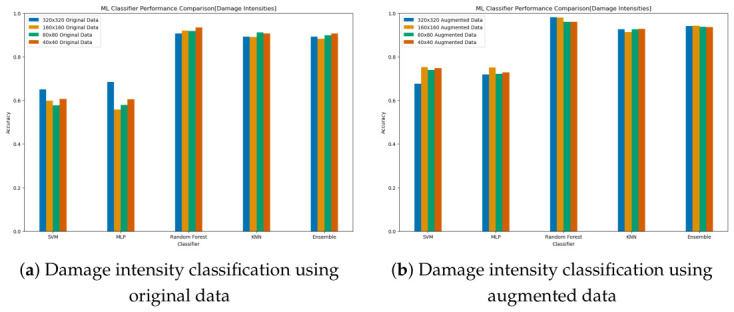
Performance comparison of traditional machine learning models for damage intensity classification using original and augmented data across different matrix sizes.

The performance accuracy was measured using classical machine learning algorithms for the seven bridge scenarios described above. These include SVM, KNN, RF, Ensemble (KNN, RF and SVM), and MLP. The model accuracy was calculated with different matrix sizes, i.e., 320 × 320, 160 × 160, 80 × 80, and 40 × 40, to better analyze classification results. The results in Figure 6a,b show the performance of these ML models regarding bridge scenarios for original data and augmented data. Similarly, Figure 7a,b represent the performance for damage intensities for original data and augmented data respectively.

From these figures, we observe that for each ML algorithm, a 40 × 40-sized matrix for the calculated feature performs the best. Among the algorithms, Random Forest performs the best in the bridge scenario classification task.

Figure 6 presents the bridge scenario classification results for original and augmented data, whereas Figure 7 presents the corresponding damage intensity classification results.

Specifically, augmented data improves performance over the original ambient data. Across classifiers, the accuracy increases with augmented data, with a notable improvement seen in the MLP, Random Forest, and Ensemble models. Random Forest achieves the highest accuracy with original and augmented ambient data. Inversely, SVM and MLP show high variance as their results are more affected by changes in the dataset. In the original ambient dataset, SVM performs relatively well, while MLP has a lower accuracy. After applying augmentation in the ambient dataset, MLP improves significantly, indicating that MLP benefits more from data augmentation than other models.

Considering the impact of matrix size during feature extraction, the smaller matrix size (40 × 40 and 80 × 80) extracted feature data performs stably across all classifiers. Among them, Random Forest performs the best. However, with sizeable-matrix-sized feature data, we see a high variance in accuracy.

The results, presented in Table 3, provide insight into the classification effectiveness of different ML models in bridge scenarios. Evaluation metrics, including accuracy, F1 score, precision, and recall, highlight the comparative strengths of these models under various conditions. Random Forest (RF) consistently emerges as the best-performing model, achieving a peak accuracy of 93.54% in the augmented dataset, confirming its robustness in bridge scenarios. The Ensemble models also demonstrate strong reliability, achieving an accuracy of 86.12%, confirming their suitability for bridge monitoring applications.

#### 6.1.2. Deep Neural Network-Based Model

**Figure 8:**
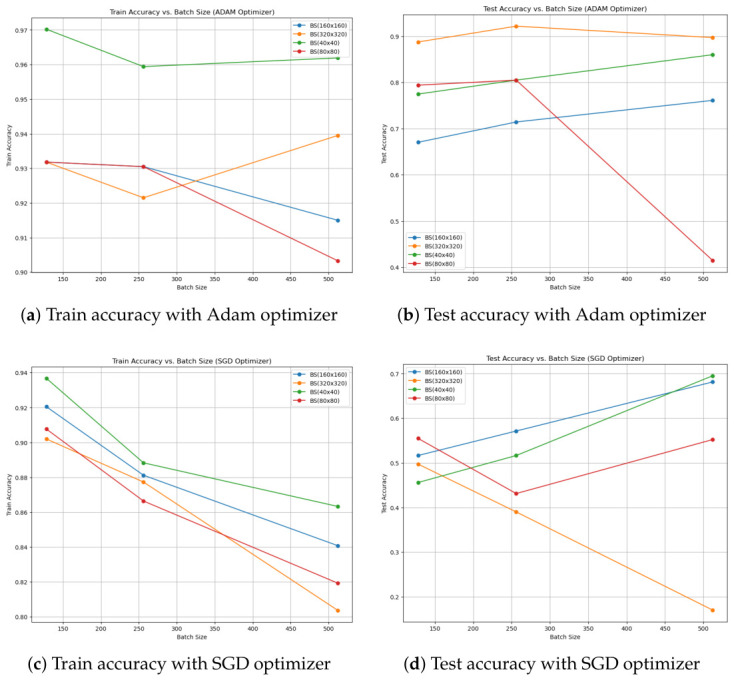
Bridge scenario classification results using Adam and SGD optimizers with learning rate 0.01 (**a**–**d**).

We also analyzed the DNN for performance evaluation. In the DNN, we tried different batch sizes, learning rates, and optimizers. The train and test accuracy results for different batch sizes using the ADAM and SGD optimizers are illustrated in Figure 8a. The batch sizes considered are 128, 256 and 512. For the ADAM optimizer, as shown in Figure 8a, the train accuracy starts high for (40 × 40) but slightly declines as the batch size increases. But for (160 × 160, 80 × 80 and 320 × 320) it fluctuates, with some showing an increasing trend and others decreasing. High training accuracy is achieved for small batches in the case of (40 × 40), but it dips initially and recovers at a larger batch size for (320 × 320) while a decline is observed for (80 × 80), which could indicate overfitting with a small batch size. Eventually (40 × 40) outperforms other experiments with any batch sizes. In test accuracy, the ADAM optimizer for (160 × 160) and BS (40 × 40) improves with increasing batch size. For (320 × 320) it shows a U shape trend, initially decreasing and recovering at higher batch sizes. For (80 × 80) it starts high but then sharply declines with a higher batch size. We see a key observation that ADAM performs well with smaller batch sizes but struggles with stability for larger ones.

However, for the Stochastic Gradient Decent (SGD) optimizer, all (40 × 40, 80 × 80, 160 × 160, and 320 × 320) show a declining trend as the batch size increases in training. Here, (40 × 40) maintains a higher accuracy, suggesting that the SGD optimizer works better with smaller batch sizes. In contrast, (320 × 320) and (80 × 80) have a lower performance, indicating that the SGD optimizer is not well suited for larger batch sizes during the training process. A similar trend is observed in the test phase. The SGD optimizer performed better with a small batch size, showing steady improvements for (40 × 40). The test accuracy deteriorates rapidly for large batch sizes, indicating that they may not work well with the SGD optimizer. Overall the SGD optimizer shows a more stable test accuracy for small batch sizes but does not generalize well to larger batches. The ADAM optimizer is more effective at converging quickly, whereas the SGD optimizer requires careful tuning to prevent a drop in accuracy.

In Figure 8, the performance of the DNN with different optimizers and batch sizes is also shown. It is evident from these figures that the train and test accuracy are higher with smaller slicing at large batch sizes with the Adam optimizer, but it is the inverse in the case of a small batch size, where this optimizer is outperformed.

The comparative analysis of ADAM and SGD for bridge scenario classification reveals that ADAM is the more effective optimizer, providing higher classification accuracy, better convergence, and better stability across all feature slice sizes. SGD, on the other hand, struggles with learning stability, particularly for larger feature slices (160 × 160 and 320 × 320), which exhibit declining accuracy trends in training and test phases. Additionally, (40 × 40) and (80 × 80) emerge as the most compelling feature slice sizes for bridge scenario classification, maintaining higher accuracy and better optimization performance than larger feature slices. SGD’s declining accuracy trends across all feature slices confirm that it is less suitable for bridge classification tasks at this learning rate. It requires further tuning and optimization strategies to improve its performance.

**Figure 9:**
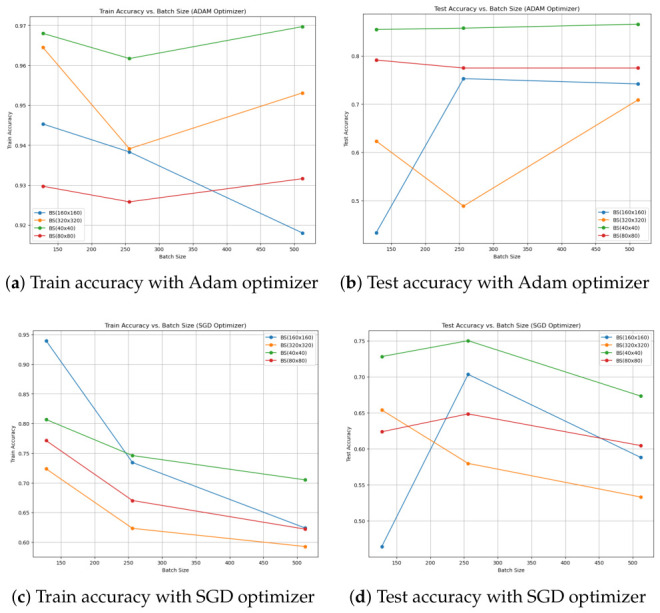
Bridge scenario classification results using Adam and SGD optimizers with learning rate 0.001 (**a**–**d**).

In conclusion, ADAM is the preferred optimizer for CNN-based bridge scenario classification at a learning rate of 0.001, as in Figure 9a,b, as it ensures higher accuracy, better convergence and better stability across varying feature slice sizes. SGD, while slightly improved with lower learning rates, remains less effective in achieving stable classification results as in Figure 9c,d.

Figure 8 presents the bridge scenario classification results for Adam and SGD optimizers using a learning rate of 0.01, while Figure 9 presents the corresponding results using a learning rate of 0.001. The findings suggest that future research should explore adaptive learning rate strategies, hybrid optimization methods, and feature selection techniques to further enhance classification accuracy and model robustness for bridge monitoring applications.

The results demonstrate that batch size selection is crucial in determining model performance. ADAM performs well with smaller batches but is less stable for larger batch sizes, while SGD is more stable but struggles with larger batch sizes. The study suggests that an optimal batch size of BS (40 × 40) or BS (160 × 160) provides the best balance between training efficiency and generalization for both optimizers.

Future research can explore the impact of different learning rate schedules and hybrid optimization strategies to further enhance the model’s robustness across varying batch sizes.

### 6.2. Damage Intensity Predictions

We have 26 different levels of bridge damage intensities divided into four subclasses. Each damage intensity represents a percentage of the damage intensity applied to our FEM.

#### 6.2.1. Traditional Machine Learning Algorithms

As explained earlier, we also compared damage intensities with traditional machine learning algorithms. The performance of machine learning (ML) classifiers for damage intensity classification was evaluated using both the original ambient dataset and the augmented dataset. The classifiers include a Support Vector Machine (SVM), a Multi-Layer Perceptron (MLP), a Random Forest, K-nearest neighbours (KNN), and an Ensemble model. Each of the 26 damage intensities described above was re-classed into four subclasses by merging. The 26 damage intensity levels were further merged into four broader damage severity classes based on the percentage of elastic modulus reduction used to define the damage intensity. These four classes were defined as low, medium, high, and critical damage. This grouping provides a compact and practically interpretable output for bridge health monitoring, allowing the predicted damage severity to be more directly related to maintenance and industrial decision-making requirements. We then assessed performance accuracy using traditional machine learning algorithms (SVM, KNN, RF, Ensemble (KNN, RF & SVM), and MLP). Each classifier was assessed using different batch sizes. We calculated the model accuracy with varying matrix sizes, that is, (320 × 320, 160 × 160, 80 × 80, and 40 × 40), to better analyze classification results. The results in Figure 7a,b show the performance of these ML models.

The results indicate that data augmentation significantly improves accuracy for most classifiers, particularly MLP and SVM, which exhibit notable improvements compared to their performance on original data. But overall, Random Forest and Ensemble models consistently outperformed other classifiers, achieving the highest accuracy across all feature data matrix sizes, with minimal impact from data augmentation, suggesting their strong generalization ability. In addition, KNN also expressed stable accuracy, but it only benefits from augmented data. Regarding sensitivity, MLP behaves with great sensitivity with data augmentation, exhibiting a substantial increase in accuracy. This suggests that the MLP model requires diverse training data with hyper-parameter fine-tuning for optimal performance. In contrast, SVM also benefits from augmented data, mainly feature data sizes 160 × 160 and 40 × 40. This reinforces the importance of feature diversity in SVM in improving generalization.

The effect of feature data slice size is also evident in all classifiers. Larger feature data slicing sizes (320 × 320 and 160 × 160) result in more stable accuracy, particularly for Random Forest, KNN, and Ensemble models. In contrast, smaller slicing sizes (80 × 80 and 40 × 40) exhibit higher variance in terms of accuracies, with MLP performing worst with small slicing when trained with original data but significantly improving with augmented data. The variability in accuracy suggests that choosing an appropriate feature data slice is crucial for classifier stability for better railway steel bridge health monitoring for improved generalization.

Table 4 provides a comparative summary of the evaluation of the performance of traditional machine learning (ML) algorithms in evaluating the severity of the damage in the health monitoring of the rail steel bridge, with different slice sizes of the feature data (320 × 320, 160 × 160, 80 × 80 and 40 × 40). Random Forest achieves the highest accuracy overall, closely followed by the Ensemble model, which remains robust regardless of data augmentation. Random Forest (RF) consistently emerges as the best-performing model, achieving the highest accuracy of 98.21% with augmented data, while Ensemble models also demonstrate robust performance, reaching an accuracy of 94.19%. However, SVM and MLP exhibit substantial improvements when augmented data is used. MLP shows the most significant performance gap between original and augmented datasets but the lowest performance for damage intensities of railway steel bridges. KNN, while performing moderately well, benefits slightly from augmentation on damage intensities but does not exhibit significant performance variations across batch sizes.

On ambient data, SVM records its lowest accuracy of 57.69% at a 80 × 80 slice size. In contrast, after augmentation, its accuracy improves to 75.27% at 160 × 160, demonstrating the effectiveness of data augmentation in improving its generalization capabilities for bridge health monitoring. Similarly, MLP, which initially struggles with an accuracy of 55.77% on ambient data, improves to 75.14% with augmented data, reinforcing the observation that deep learning models require diverse and enriched data for optimal learning in structural health assessment.

In conclusion, the Ensemble and Random Forest classifiers are the most effective models for classifying damage intensity, offering the highest accuracy and stability across batch sizes. However, MLP and SVM greatly benefit from data augmentation, highlighting the importance of training data diversity. The study further confirms that batch size selection is crucial in achieving stable and high-performing ML models, with larger batch sizes (320 × 320, 160 × 160) leading to better generalization. Future research could explore hybrid feature extraction methods and adaptive learning rate schedules to optimize classification performance.

#### 6.2.2. Deep Neural Network

The accuracy plots provide information on the performance comparison of the SGD and ADAM optimizers in classifying damage intensities in railway steel bridges using learning rates of 0.01 and 0.001. The models were trained with different feature data slice sizes and evaluated based on training and test accuracy trends.

**Figure 10:**
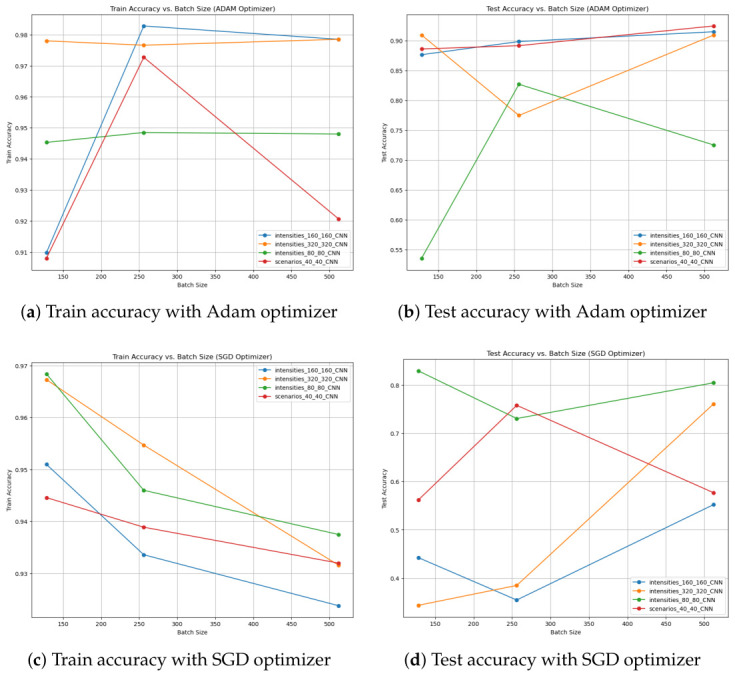
Damage intensity classification results using Adam and SGD optimizers with learning rate 0.01 (**a**–**d**).

The comparative analysis of ADAM and SGD optimizers for the classification of damage intensities in railway steel bridges was carried out using a learning rate of 0.01, with varying feature data slice sizes (320 × 320, 160 × 160, 80 × 80 and 40 × 40). The test and training accuracy trends were evaluated to assess the optimizers’ performance and generalization capability. The test accuracy results indicate that ADAM consistently outperforms SGD in all feature data slice sizes as in Figure 10a,c, demonstrating better convergence and stability. The test accuracy trends reveal that the feature data slice (160 × 160) achieves the highest and most stable accuracy under ADAM, as shown in Figure 10b, maintaining a progressive improvement as batch size increases. Similarly, DI (320 × 320) maintains a stable upward trend, confirming ADAM’s ability to generalize well across different bridge monitoring configurations. However, DI (40 × 40) and DI (80 × 80) exhibit fluctuating trends, with DI (40 × 40) showing an initial rise before declining at larger batch sizes, indicating possible overfitting with smaller feature slice sizes.

In contrast, the SGD optimizer struggles with stability and generalization, as reflected in the test accuracy results. Unlike ADAM, SGD experiences inconsistent accuracy trends, particularly for (160 × 160) and (320 × 320), where accuracy starts lower and increases at larger batch sizes. However, (40 × 40) and (80 × 80) show erratic behavior, with a noticeable decrease at specific batch sizes, as seen in Figure 10c,d, further suggesting the sensitivity to feature slice size variations of the SGD optimizer. The lower initial accuracy and unstable trends indicate that SGD requires more fine-tuning to provide effective learning in bridge damage localization and classification.

**Figure 11:**
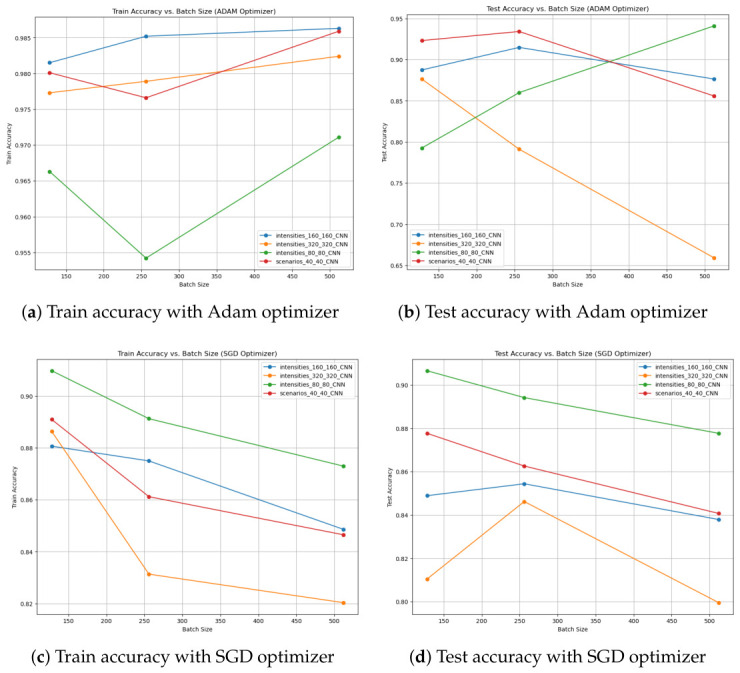
Damage intensity classification results using Adam and SGD optimizers with learning rate 0.001 (**a**–**d**).

Figure 10 presents the damage intensity classification results for Adam and SGD optimizers using a learning rate of 0.01, while Figure 11 presents the corresponding results using a learning rate of 0.001. The training accuracy results further highlight the differences between ADAM and SGD. Under ADAM, the training accuracy remains high for all feature slice sizes, with (160 × 160) and (80 × 80) reaching peak accuracy near 98%, confirming strong convergence. DI (40 × 40) maintains a stable accuracy trend, with minimal fluctuations, while (320 × 320) shows a slight decline at larger batch sizes, suggesting that regularization may be needed to mitigate overfitting. In contrast, SGD exhibits declining training accuracy trends across all feature slice sizes, reinforcing its instability at this learning rate. The gradual decrease in training accuracy with increasing batch size indicates SGD’s difficulty maintaining effective learning, making it less suitable for this application.

The comparative evaluation of ADAM and SGD optimizers for damage classification and localization in railway steel bridges was conducted using two learning rates (0.01 and 0.001) and feature data slice sizes of 320 × 320, 160 × 160, 80 × 80 and 40 × 40. The test and training accuracy trends were analyzed to assess both optimizers’ optimization performance, generalization capability, and localization accuracy. The results indicate that ADAM consistently outperforms SGD, achieving higher accuracy and stability across all configurations. ADAM maintains a stable and increasing trend in test accuracy, with (160 × 160) and (80 × 80) reaching peak accuracies above 90%, demonstrating effective damage localization and classification capabilities. However, (40 × 40) exhibits fluctuating accuracy, initially increasing and declining at larger batch sizes, suggesting overfitting issues when using extremely fine-grained feature slices. The (320 × 320) feature slice shows a decreasing trend, indicating that larger feature slices may generalize too broadly, leading to reduced classification precision.

In contrast, SGD exhibits lower test accuracy and greater instability between feature slice sizes. The test accuracy trends for (160 × 160) and (320 × 320) under SGD show inconsistent improvements, while (40 × 40) and (80 × 80) demonstrate erratic behavior, failing to maintain performance consistency. At a learning rate of 0.01, SGD struggles with generalization, resulting in poor classification accuracy and significant performance variations between batch sizes. However, when the learning rate is reduced to 0.001, SGD shows slight improvements in stability, although it remains inferior to ADAM in overall accuracy and robustness.

The training accuracy trends further highlight the differences between the two optimizers. Under ADAM, as in Figure 10a and Figure 11a, the training accuracy remains consistently high, with (160 × 160) and (80 × 80) achieving nearly 98% accuracy, confirming strong convergence and practical learning. Although (40 × 40) initially dips and later recovers, its fluctuating trend indicates overfitting concerns at smaller feature slice sizes. Meanwhile, (320 × 320) maintains stable but slightly declining accuracy at larger batch sizes, suggesting that larger feature slices may be less effective for precise damage localization. In contrast, SGD exhibits declining training accuracy trends for all feature slice sizes, emphasising its difficulty in maintaining learning stability. As batch sizes increase, SGD struggles to maintain training accuracy, with (160 × 160) and (320 × 320) showing the most severe drops, confirming its inability to achieve stable convergence at higher learning rates. Although reducing the learning rate to 0.001 slightly improves the stability of the SGD optimizer, it still fails to reach the same accuracy levels as ADAM.

The comparative findings confirm that ADAM is the superior optimizer for CNN-based damage classification and localization in railway steel bridges, as it ensures better convergence, higher accuracy, and greater stability for different feature slice sizes. In contrast, SGD struggles with training convergence and test accuracy stability, particularly at higher learning rates. The results also indicate that (160 × 160) is the most reliable feature slice size for classification and localization, balancing granularity and generalization. The size (40 × 40) demonstrates inconsistent behavior with both optimizers, suggesting that tiny feature slices may introduce noise rather than enhance localization precision. Furthermore, (320 × 320) performs relatively well under ADAM but suffers from declining accuracy under SGD, reinforcing the limitations of SGD in optimizing large feature representations for damage classification.

Overall, the findings suggest that ADAM is the preferred optimizer for damage classification and localization in railway steel bridges, as it provides higher accuracy, better convergence, and better stability across different feature slice sizes and learning rates. SGD, while slightly improved at a lower learning rate of 0.001, remains less effective in achieving stable and accurate classification results. These results indicate that future research should focus on adaptive learning rate strategies, hybrid optimization methods, and feature selection techniques to improve classification accuracy and localization precision in railway bridge health monitoring.

## 7. Conclusions

This paper has proposed a practical implementation of the machine learning method to classify various damage scenarios, in their location and intensity, of an in-service steel railway bridge. The framework is based on a deep neural network classifier, trained with features extracted from acceleration data produced by a physics-based reduced-order model of the bridge. In observation, it becomes evident that with smaller slicing, higher accuracy is achieved with both standard and augmented data. Further work has been carried out to introduce four class damage intensities within the standard range and train and test this model in various scenarios using a DNN model.

## 8. Declaration

Authors occasionally used generative AI tools to improve the readability of some parts of the text; they reviewed the content and take full responsibility for the paper’s content.

## Figures and Tables

**Table 1:** Damage scenario descriptions. The locations of damaged elements are shown in Figure 1 and Figure 2.

Scenario	Description
S1	Braces, degradation due to bending mechanisms.
S2	Transverse member, degradation due to bending mechanisms, single transverse at midspan.
S3	Transverse member, degradation due to bending mechanisms, three transverses at midspan.
S4	Braces, degradation due to shear mechanisms.
S5	Transverse member, degradation due to shear mechanisms, transverse members at the header.
S6	Left pier, degradation due to vertical failure.
S7	Piers, degradation due to vertical failure of both piers.

**Table 2:** Architecture and hyper-parameters of the proposed model.

Category	Parameter	Value/Description
Hyper-parameter	Learning Rate	0.01, 0.001
	Batch Size	128, 264, 512
	Epochs	1000
	Optimizer	Adam, SGD
	Loss Function	Cross-Entropy Loss
	Input Shape	(6, 128)
Model Architecture	Fully Connected Layers	4
	Activation Function	ReLU, GELU
	Output Layer	Softmax (10 & 4 classes)

**Table 3:** Performance comparison of traditional machine learning algorithms for bridge scenario classification. The best performers are in bold.

Dataset	ML Algorithm	Accuracy (%)	F1 Score (%)	Precision (%)	Recall (%)
**Ambient Data**	KNN-320	64.28	64.03	64.58	64.29
	KNN-160	68.13	68.31	69.28	68.13
	KNN-80	66.21	66.41	67.06	66.21
	KNN-40	**72.80**	**73.14**	**73.87**	**72.80**
	RF-320	76.65	76.72	77.03	76.65
	RF-160	76.37	76.64	77.47	76.37
	RF-80	75.27	75.42	75.71	75.27
	RF-40	**83.79**	**83.99**	**84.32**	**83.79**
	SVM-320	50.00	43.73	57.02	50.00
	SVM-160	57.14	51.88	54.51	57.14
	SVM-80	**59.06**	56.36	64.92	**59.07**
	SVM-40	57.69	**56.38**	**66.79**	57.69
	MLP-320	44.51	41.03	47.77	44.51
	MLP-160	**57.42**	**53.76**	**58.93**	**57.42**
	MLP-80	31.59	28.88	28.01	31.59
	MLP-40	38.18	33.20	57.50	38.19
	Ensemble-320	70.05	69.29	74.58	70.05
	Ensemble-160	71.15	70.73	75.51	71.15
	Ensemble-80	70.60	70.83	74.70	70.60
	Ensemble-40	**78.02**	**78.43**	**91.99**	**78.02**
**Augmented Data**	KNN-320	75.41	75.47	75.78	75.42
	KNN-160	77.47	77.57	77.85	77.47
	KNN-80	76.24	76.33	76.47	76.24
	KNN-40	**79.39**	**79.39**	**79.60**	**79.39**
	RF-320	**93.54**	**93.52**	**93.58**	**93.54**
	RF-160	92.72	92.73	92.77	92.72
	RF-80	93.27	93.29	93.38	93.27
	RF-40	92.99	93.03	93.23	92.99
	SVM-320	42.03	38.03	56.78	42.03
	SVM-160	**63.33**	61.28	**70.46**	**63.32**
	SVM-80	61.81	56.66	58.32	61.81
	SVM-40	63.32	**62.74**	69.46	**63.32**
	MLP-320	55.91	51.86	62.71	55.91
	MLP-160	60.99	60.60	68.07	60.98
	MLP-80	58.10	58.51	66.57	58.11
	MLP-40	**61.40**	**61.44**	**69.54**	**61.40**
	Ensemble-320	81.73	82.01	85.35	81.73
	Ensemble-160	82.97	83.24	86.25	82.96
	Ensemble-80	83.52	83.51	86.60	83.51
	Ensemble-40	**86.12**	**86.37**	**88.90**	**86.13**

**Table 4:** Performance comparison of traditional machine learning algorithms for damage intensity classification. The best performer are in bold.

Dataset	ML Algorithm	Accuracy (%)	F1 Score (%)	Precision (%)	Recall (%)
**Ambient Data**	KNN-320	89.28	88.98	90.28	89.89
	KNN-160	89.01	88.95	90.03	89.01
	KNN-80	**91.21**	**91.14**	**91.74**	**91.20**
	KNN-40	90.66	90.56	90.89	90.65
	RF-320	90.66	90.46	91.02	90.65
	RF-160	92.03	91.91	92.14	92.03
	RF-80	91.76	91.61	92.05	91.75
	RF-40	**93.41**	**93.31**	**93.50**	**93.49**
	SVM-320	**65.10**	**61.61**	**76.17**	**65.11**
	SVM-160	59.89	51.18	50.32	59.89
	SVM-80	57.69	47.86	47.55	57.69
	SVM-40	60.71	53.68	67.67	60.71
	MLP-320	**68.41**	**63.49**	**74.38**	**68.40**
	MLP-160	55.77	46.27	46.52	55.76
	MLP-80	57.96	49.06	51.24	55.76
	MLP-40	60.44	53.33	59.58	60.43
	Ensemble-320	89.28	88.96	91.13	89.30
	Ensemble-160	88.18	88.13	89.96	88.18
	Ensemble-80	89.83	89.72	90.98	89.84
	Ensemble-40	**90.66**	**90.52**	**91.68**	**90.65**
**Augmented Data**	KNN-320	92.71	92.64	92.71	92.72
	KNN-160	91.34	91.32	91.34	91.35
	KNN-80	92.58	92.56	92.69	92.58
	KNN-40	**92.85**	**92.82**	**92.93**	**92.85**
	RF-320	**98.21**	**98.21**	**98.24**	**98.21**
	RF-160	98.07	98.07	98.08	98.07
	RF-80	96.02	95.98	96.18	96.01
	RF-40	96.01	95.99	96.04	96.02
	SVM-320	67.58	62.93	78.47	67.58
	SVM-160	**75.27**	**72.37**	**81.66**	**75.27**
	SVM-80	73.91	71.07	79.35	73.90
	SVM-40	74.86	71.79	81.14	74.86
	MLP-320	71.84	68.41	72.91	71.84
	MLP-160	**75.14**	**73.64**	**75.85**	**75.13**
	MLP-80	72.25	71.91	72.10	72.25
	MLP-40	72.80	68.99	72.12	72.80
	Ensemble-320	94.09	94.10	94.49	94.09
	Ensemble-160	**94.19**	**94.19**	**94.82**	**94.23**
	Ensemble-80	93.81	93.76	94.38	93.82
	Ensemble-40	93.54	93.47	94.06	93.54

## Data Availability

Raw data is covered by a Non-Disclosure Agreent by the industrial partner. Augmented data produced in our laboratory can be requested by email to the corresponding authors.
